# Metabolomic Analysis of Antifungal Secondary Metabolites from *Achaetomium sophora* HY17 in Co-Culture with *Botrytis cinerea* HM1

**DOI:** 10.3390/microorganisms13122794

**Published:** 2025-12-08

**Authors:** Guanlan Liu, Zhiyun Tang, Ruotong Wang, Ying Xin, Peiwen Gu

**Affiliations:** 1 College of Forestry and Prataculture, Ningxia University, Yinchuan 750021, China; 17609572555@163.com; 2 School of Agriculture, Ningxia University, Yinchuan 750021, China; tzy183522487@163.com (Z.T.); xinying0433@126.com (Y.X.); 3 State Key Laboratory for Crop Stress Resistance and High-Efficiency Production, College of Plant Protection, Northwest A&F University, Yangling 712100, China; wangruotong0729@163.com

**Keywords:** *Endophytic fungi*, *Achaetomium*, *Botrytis cinerea*, co-culture, metabolomics

## Abstract

Secondary metabolites produced by endophytic fungi living in medicinal plants are important resources in the field of biological control. In this study, *Achaetomium sophora* HY17, an endophytic fungus of *Sophora alopecuroides*, was taken as the research object and *Botrytis cinerea* HM1 as the target pathogen, and the response characteristics and antifungal mechanism of secondary metabolites produced during their interaction were explored through a co-culture system combined with metabonomic analysis. The key findings are as follows: (1) *A. sophora* HY17 produces many kinds of secondary metabolites, such as alkaloids, flavonoids, and phenolic acids, among which 10 different metabolites, such as Sophoridine, Matrine, and Luteolin, are significantly up-regulated during the interaction process and are the core antifungal active substances; (2) KEGG pathway enrichment analysis revealed that the phenylalanine metabolic pathway was significantly enriched during the interaction between the two fungi, and the activation of this pathway was the key regulatory mechanism underlying *A. sophora* HY17′s ability to cope with pathogen stress and synthesize antifungal metabolites. This study reports *A. sophora* HY17 as a new species, confirms its broad application prospects as a multifunctional and efficient biocontrol strain, and provides a core theoretical basis and target direction for mining antifungal substances from endophytic fungi to develop new biocontrol agents.

## 1. Introduction

Microbial metabolites are valuable resources for discovering new antifungal compounds in biological pest control investigations [1]. Endophytic fungi living in medicinal plants have gained attention as novel microbial sources in recent years. The secondary metabolites of endophytic fungi in most medicinal plants contain bioactive substances that can inhibit pathogenic fungi, making them potential valuable materials for discovering new antifungal agents [2]. For instance, Chen et al. [3] discovered two new sesquiterpenoids—leptosphin A and B—in the solid fermentation culture of *Leptosphaeria* sp. XL026, an endophytic fungus living in the leaves of the medicinal plant *Panax notoginseng*. Leptosphin B exhibited significant inhibitory activity against *Bacillus subtilis*. Therefore, the synthesis of active metabolites by endophytic fungi in medicinal plants provides a significant resource for identifying new compounds with antifungal activities, and screening these secondary metabolites for effective antifungal compounds is of significance [4].

In view of the huge metabolic potential of endophytic fungi, how to effectively stimulate their biosynthesis ability has become a research hotspot. In recent years, fungal co-culture has become commonly accepted as a promising approach for discovering novel antimicrobial metabolites [5]. Studies have revealed that most microorganisms’ gene clusters remain inactive when cultured individually. However, the co-culture strategy can activate these silent genes by altering the external conditions with the necessary cofactors. This activation promotes the production of numerous secondary metabolites with unique structures and significant physiological activities [6]. To some extent, microbial co-culture simulates the complex living conditions of microorganisms in their natural environment [7]. This simulation utilizes interactions such as survival competition between multiple microorganisms to stimulate the production of metabolites in respect to both diversity and quantity [8]. The differential metabolites synthesized by microorganisms in a co-culture are closely associated with their chemical defense mechanisms and often possess various biological activities, such as antifungal and cytotoxic effects [9,10]. There are multiple successful examples of using the microbial co-culture approach to explore the potential synthesis of microbial secondary metabolites [11]. Liu et al. [12] discovered that when *Aspergillus oryzae* and *Zygosaccharomyces rouxii* were co-cultured, *A. oryzae* produced Taurine, Imidazoleacetic acid, and other substances. These substances significantly inhibited the growth of *Z. rouxii*, indicating that fungal co-culture can impact the yield and type of secondary metabolites.

In order to tap into more diverse antifungal resources using the co-culture strategy, our research focuses on fungal groups whose metabolic potential has not been fully developed. *Achaetomium fungi* are one of those fungal groups. Fungi belonging to the *Achaetomium* genus are abundant and can be found in various decaying plant residues, fibrous materials like paper and wood, seeds, leather, animal excreta, and plant tissues [13]. They have wide applications in the food industry, husbandry, and other agriculture fields. So far, studies have found that the metabolites of fungi belonging to the *Achaetomium* genus mainly contain cell wall degrading enzymes like mannanase [14,15] and polygalacturonase [16,17]. Mannanase is a crucial hemicellulase that is widely utilized in husbandry, paper making, detergent production, food processing, and oil extraction. Polygalacturonase is a pectinase and has extensive applications in fruit juice production, paper making, textile industry, and oil extraction. Therefore, *Achaetomium fungi* have gained increasing attention in industrial production and scientific research; however, limited studies have been conducted on their biological control.

Based on this, *Achaetomium* sp. Hy17, an endophytic fungus with outstanding biocontrol potential, was selected in this study. *Sophora alopecuroides* is an important medicinal plant, and its whole grass, roots, and seeds can be used as medicine. The secondary metabolites found in *Sophora alopecuroides* are mainly alkaloids and flavonoids. *Achaetomium* sp. HY17 isolated from healthy wild *Sophora alopecuroides* plants collected in Huamachi Town (107.46° E, 37.77° N, 1336.4 m above sea level), Yanchi County, Ningxia Hui Autonomous Region, had been found to produce metabolites with antifungal activity similar to that of the host plant, indicating that it is a biocontrol strain with great application prospects [18,19]. However, at present, the antifungal activity, production mechanism, and action of its antifungal substances remain unclear. This study aimed to clarify the broad-spectrum antifungal activity of *Achaetomium* sp. HY17. The study investigated dynamic changes in the secondary metabolites of *Achaetomium* sp. HY17 during its interaction with *B. cinerea* HM1 using the co-culture approach and metabolomic analysis to discover antifungal secondary metabolites. This research provides a theoretical foundation for designing and developing biocontrol agents.

## 2. Materials and Methods

### 2.1. Test Strain and Standard Substances

The biocontrol fungus *Achaetomium* sp. HY17 strain (CGMCC No.41060) was deposited at the China General Microbiological Culture Collection Center, Beijing, China.

The species of pathogenic fungi used in this study, their sources of isolation, and the methods of identification are detailed in Table S2.

The standard test products, including Sophoridine (HPLC ≥ 98%), Sophocarpine (HPLC ≥ 98%), Matrine (HPLC ≥ 98%), Oleoylethanolamine (HPLC ≥ 90%), 3-Hydroxyanthranilic acid (10 mM in DMSO), Chalcone (HPLC ≥ 98%), Cynaroside (HPLC ≥ 98%), Daidzein (HPLC ≥ 95%), Vitexin (HPLC ≥ 98.5%), and 1-Tetralone (HPLC ≥ 98%), were purchased from Shanghai Yuanye Biotechnology Co., Ltd. (Shanghai, China).

### 2.2. Evaluation of Antifungal Activity

The in-dish confrontation method [20] was used to inoculate the mycelial disks of *Achaetomium* sp. HY17 and pathogenic fungi (5 mm in diameter) on PDA medium in 90 mm Petri dishes, with the two mycelial disks placed 4 cm apart. Two experimental treatments were conducted: simultaneous inoculation of biocontrol and pathogenic fungi, and delivery of fungal control for 3 days prior to pathogenic fungal inoculation. Three replicates were set up for each treatment. The Petri dishes were incubated at 25 °C in alternating light and dark conditions, and inoculation with pathogenic fungi only served as the blank control. When the pathogenic fungi in the blank control group almost filled the Petri dish, measurements of the colony radius (Rc) and the extension of colony growth towards the antifungal control (R) in the experimental group were taken, from which the inhibition rate was calculated. Statistical software programs Excel 2003 and SPSS 19.0 were used to analyze the difference. A one-way ANOVA was used for comparison between groups.

### 2.3. Classification Status Identification of Strain HY17

Strain HY17 was inoculated on the PDA culture medium and cultured at 28 °C in alternate light and dark conditions and at 28 °C in dark conditions. According to the classification method of fungi, morphological identification was conducted based on strain colony characteristics such as shape, texture, and color [21]. A fungal genomic DNA extraction kit was used to extract DNA for molecular identification. Primers ITS1/ITS4 [22], LROR/LR3 [23], and Bt2a/Bt2b [24] were designed for PCR amplification reactions targeting the internal transcription spacers 1 and 2 of fungal 5.8S nrDNA, the D1/D2 domain of 28S nrDNA, and the β-tubulin gene sequence, respectively. The PCR system (25 µL) consisted of a master mix (12.5 µL), DNA (2 µL), and forward and reverse primers (1 µL each), supplemented with ddH_2_O. The PCR conditions included 34 cycles of predenaturation at 94 °C for 3 min, followed by denaturation at 94 °C for 30 s, annealing at 55 °C for 30 s, and extension at 72 °C for 1 min. A final extension was performed at 72 °C for 10 min. The PCR products were detected using a spectrophotometer and analyzed with agarose gel electrophoresis (1%). Subsequently, the samples were sent to Sangon Bioengineering (Shanghai) Co., Ltd. (Shanghai, China), for sequencing. Sequence information with high homology was searched and downloaded from the NCBI BLAST database (https://blast.ncbi.nlm.nih.gov/Blast.cgi accessed on 2 November 2023). MEGA7 software and the Neighbor-Joining method were employed to construct the phylogenetic tree [25]. The Muscle algorithm in MEGA7 software was used to accurately compare the sequences of HY17 and the closest model strain. Then, using the sequence distance calculation function of MEGA7, the percentage of sequence difference in pairs was calculated. The sequences obtained from the molecular identification of HY17 are presented in Table S5.

### 2.4. Co-Culture Sample Collection

The fungi *A. sophora* HY17 and *B. cinerea* HM1 were co-cultured on PDA medium according to Triastuti et al.’s method [26], with their mycelial disks placed 4 cm apart, and incubated at 25 °C in alternating light and dark conditions. At 3 dpi, 6 dpi, and 10 dpi when the mycelium of the two fungi made contact, a sterilized slide was used to scrape off 1 g of the mycelium from the co-culture (CO) area, which was then transferred into a 5 mL frozen storage tube as one sample. This process was performed in triplicate. Blank controls were prepared by culturing colonies of *A. sophora* HY17 (Ch) and *B. cinerea* HM1 (HM) separately under identical culture conditions. The collected mycelium samples were labeled as Ch-3d, HM-3d, CO-3d, Ch-6d, HM-6d, CO-6d, Ch-10d, HM-10d, and CO-10d (numbers represent days of cultivation). Store at −80 °C for later use.

### 2.5. UPLC-MS/MS Detection and Analysis

#### 2.5.1. Sample Extraction

Remove the sample from the −80 °C refrigerator to thaw (See Section 2.4 for sample collection.). Vortex for 1 min, or if uniform vortexing is not possible, stir manually with a weighing spoon for 30 s. Proportionally add 70% methanolic water internal standard extract pre-chilled at −20 °C (600 μL extractant per 50 mg sample) and vortex for 15 min. Centrifuge (15,616× *g*, 4 °C) for 3 min, take the supernatant and filter it by a microporous membrane (0.22 μm), then save it in the injection bottle for LC-MS/MS test.

#### 2.5.2. UPLC-MS/MS Detection

The sample extracts were analyzed using an UPLC-ESI-MS/MS system (UPLC, ExionLC™ AD, https://sciex.com.cn/; MS, Applied Biosystems 6500 Q TRAP, https://sciex.com.cn/, SCIEX Shanghai Aibo Caisi Analytical Instrument Trading Co., Ltd. Shanghai, China). The analytical conditions were as follows: UPLC with an Agilent SB-C18 column (1.8 µm, 2.1 mm × 100 mm); the mobile phase consisted of solvent A, composed of pure water with 0.1% formic acid, and solvent B, composed of acetonitrile with 0.1% formic acid. Sample measurements were performed with a gradient program that employed the starting conditions of 95% A and 5% B. Within 9 min, a linear gradient to 5% A/95% B was programmed, and the composition of 5% A and 95% B was maintained for 1 min. Subsequently, the composition was adjusted to 95% A and 5.0% B within 1.1 min and maintained for 2.9 min. The flow velocity was set at 0.35 mL per minute; the column oven was set to 40 °C; and the injection volume was 2 μL. The effluent was alternatively connected to an ESI-triple quadrupole-linear ion trap (QTRAP)-MS. Representative metabolites Q1 and Q3 are shown in Supplementary Table S3. This part of the study was conducted by Wuhan Maiwei Metabolic Biotechnology Co., Ltd. (Wuhan, China).

#### 2.5.3. Statistical Data Analysis

A quality control (QC) sample was prepared by mixing the sample extracts, which was used to analyze the repeatability of samples under the same treatment method. In the process of instrument analysis, a quality control sample is usually inserted for every 10 detection and analysis samples to monitor the repeatability of the analytical process. By overlapping and displaying the total ion current (TIC) diagrams of different QC samples, we can judge the repeatability of metabolite extraction and detection, that is, technical repetition. The high stability of the instrument provides an important guarantee for the repeatability and reliability of data. The CV value stands for the Coefficient of Variation, which is the ratio of the standard deviation to the average of the original data, and reflects the degree of data dispersion. The empirical cumulative distribution function (ECDF) can be used to analyze the frequency of the CV of substances relative to a reference value. The higher the proportion of substances with a lower CV value in the QC samples, the more stable the experimental data.

The qualitative analysis of metabolites was based on the self-built database MWDB of Maiteville Biotechnology Co., Ltd. (Wuhan, China). This database was constructed by using a large number of commercial real standards and collecting their accurate retention time and secondary mass spectra under the same LC-MS/MS platform conditions as this study. The identification of metabolites in the experimental samples needed to meet the retention time and the high matching between the secondary mass spectrum and the information of corresponding standards in the database. After identification, the multiple reaction monitoring (MRM) mode of triple quadrupole mass spectrometry was used to quantify each metabolite. The metabolite data obtained from different samples were subjected to qualitative and quantitative analyses using mass spectrometry (Analyst 1.6.3). The data were normalized; annotated based on retention time, plasma–nucleus ratio, and peak intensity; and further confirmed using corresponding standards for most substances. Pearson correlation coefficient, or r, was used as the evaluation index of biological repetition correlation and calculated with the built-in cor function of R software (base package; Hmisc. 3.5.1; 4.4.0). The closer |r| is to 1, the stronger the correlation between two duplicate samples. Multidimensional statistics were employed to establish a stable and reliable mathematical model for metabolite analysis. Firstly, Principal Component Analysis (PCA) with unsupervised pattern recognition was conducted to preliminarily examine the overall metabolite differences between sample groups and within-group variations among samples. Partial least squares discriminant analysis (PLS-DA), a supervised pattern recognition method, was utilized to identify differential metabolites and determine the overall differences between groups. Orthogonal partial least squares discriminant analysis (OPLS-DA) was employed to eliminate irrelevant differences and identify differential metabolites among the sample groups. This model carries out 200 random permutation and combination experiments on the data. The selection criterion for differential metabolites was based on a variable importance in projection (VIP) score greater than 1. Model evaluation and analysis were based on the R^2^X, R^2^Y, and Q^2^ parameters. A higher value closer to 1 indicates a more stable and reliable model. Differential metabolites were screened using VIP values > 1, Student’s *t*-test with *p* < 0.05, and fold-change measurements. Metabolite differences were determined by applying Log2 transformation with VIP > 1 and *p* < 0.05 thresholds. Metabolites with Log2FC ≥ 2 or Log2FC ≤ 0.5 were considered to be differential metabolites. The KEGG PATHWAY database provides information on molecular interactions, physiological reactions, biochemical processes, and gene–product relationships in manually mapped metabolic pathways. By aligning metabolite information with KEGG compound IDs, we can obtain insights into the involved metabolic pathways and evaluate their impact on biological processes. The analytical software programs used in this study are listed in Table S4. This part of the study was conducted by Wuhan Maiwei Metabolic Biotechnology Co., Ltd.

### 2.6. Determination of the Inhibitory Activity of Differential Metabolites

Based on the differential metabolites obtained in the screening process described above, the standard substances of 10 selected metabolites (listed in Section 2.1) were purchased, and their antifungal activities were evaluated. According to the method of Liu et al. [27], the experiment setup included a drug group and a blank control group. In the drug group, each standard was dissolved in methanol, and the mother liquor with a concentration of 50 mg/mL, 100 mg/mL, 150 mg/mL, 200 mg/mL, 250 mg/mL, and 300 mg/mL was prepared in turn and filtered using a 0.22 μm microporous membrane for later use. When preparing the culture medium containing the standards, 10 μL of the mother liquor was added to each dish and mixed with 10 mL of PDA culture medium so that the final concentration of the standards corresponded to 50, 100, 150, 200, 250, and 300 μg/mL, respectively. The same volume of methanol was added to the blank control group. After the culture medium was placed on an ultra-clean workbench, the liquid surface was blown by sterile airflow for at least 4 h, and the culture medium was kept sterile for one night; *B. cinerea* HM1 mycelial disks with a diameter of 5 mm were inoculated in the center of each plate and cultured at 25 °C under alternating light and dark conditions. When the colony in the control group had grown to almost cover the full plate, the diameter of the colony was measured using the cross method, and the bacteriostatic rate was calculated according to the following formula: inhibition rate (%) = [(colony diameter of blank control group-colony diameter of drug group)/colony diameter of blank control group] × 100. Finally, Prism 9 was used to analyze the data, with the regression equation fitted using the nonlinear least square method, and EC_50_ and EC_90_ were calculated.

### 2.7. A. sophora HY17 Extraction and Determination of Alkaloids and Flavonoids

#### 2.7.1. Extraction of Alkaloids and Flavonoids from Samples

The isolated and purified strain was inoculated in PDA medium. After 7 days of culture at 26 °C, 5 mycelial disks with a diameter of 0.8 cm were inoculated in 150 mL PDB medium. After 7 days of culturing at 26 °C and shaking at 175 r/min, the mycelia were collected via filtration with 4 layers of sterile mesh and dried to a constant weight in an oven at 50 °C. A total of 1 g of each mycelium was weighed. After being ground to powder in a mortar, the mycelium was placed in a 50 mL centrifuge tube and 15 mL methanol was added; the solution was mixed evenly, sonicated at 45 °C for 60 min, and centrifuged at 9500 r/min for 10 min; finally, the supernatant was evaporated and concentrated to 2 mL on a rotary evaporator after passing through a 0.45 μm filter, and stored at 4 °C until examined.

#### 2.7.2. Detection of Alkaloids by HPLC

According to the method of Ju et al. [19], the chromatographic conditions were as follows: ultimate AQ-C18 (4.6 μm × 250 mm × 5 mm); the mobile phase consisted of 0.2% phosphoric acid (triethylamine 2.00 mol/L; A) and acetonitrile (B), with an elution gradient of 0~22 min and 96.5% A; the mobile phase composition was maintained for 22~23 min with 96.5%~0% A; 23~27 min with 100% A; 27~28 min with 0%~96.5% A; and 28~34 min with 96.5% A; the volume of the sample was 10 μL, the flow rate was set to 1.0 mL/min, the detection wavelength was 205 nm, and the column temperature was 30 °C. By comparing the HPLC chromatogram of the endophytic fungus sample to be detected with that of the alkaloid standard, it can be determined that the sample contains the corresponding alkaloid if the sample has a peak with the same or similar retention time as that of the standard.

#### 2.7.3. Detection of Flavonoids by HPLC

According to the method of Li et al. [28], the chromatographic conditions were as follows: ultimate AQ-C18 (4.6 μm × 250 mm × 5 mm); mobile phase A was composed of acetonitrile and mobile phase B was composed of 0.8% phosphoric acid aqueous solution, with an elution gradient of 0~5 min and 18% A; the mobile phase composition was maintained for 5~8 min with 24% A; 8~10 min with 36% A; 10~20 min with 38% A; 20~20.01 min with 45% A; 20.01~25 min with 45% A; 25~30 min with 65% A; 30~35 min with 70% A; 35~36 min with 71% A; and 36~38 min with 18% A; the injection volume was 10 μL, the flow rate was 0.8 mL/min, the detection wavelength was 225 nm, and the column temperature was 25 °C. By comparing the HPLC chromatogram of the endophytic fungus sample to be detected with that of the flavonoid standard, it can be determined that the sample contains the corresponding flavonoid if the sample has a peak with the same or similar retention time as that of the standard.

## 3. Results

### 3.1. Evaluation of Antifungal Activity of Achaetomium sp. HY17

The fungal antagonism results observed on Petri dishes showed that when *Achaetomium* sp. HY17 was inoculated with pathogenic fungi, its inhibition effect on *B. cinerea* HM1 was the best, with an inhibition rate of 74.60, followed by *C. siamese* NX2-7, with an inhibition rate of 68.08%. If *Achaetomium* sp. HY17 was inoculated three days in advance prior to inoculation with pathogenic fungi, its inhibitory effects on the seven tested pathogenic fungi were significantly enhanced, especially on *B. cinerea* HM1, *R. solani* pn5-2, *F. avenacea* YM1, and *C. siamense* NX2-7, with the inhibition rates all exceeding 90% (Table 1). Therefore, strain HY17 is a promising biocontrol agent with broad-spectrum antifungal activity.

### 3.2. Classification and Identification of Achaetomium sp. HY17

**Figure 1:**
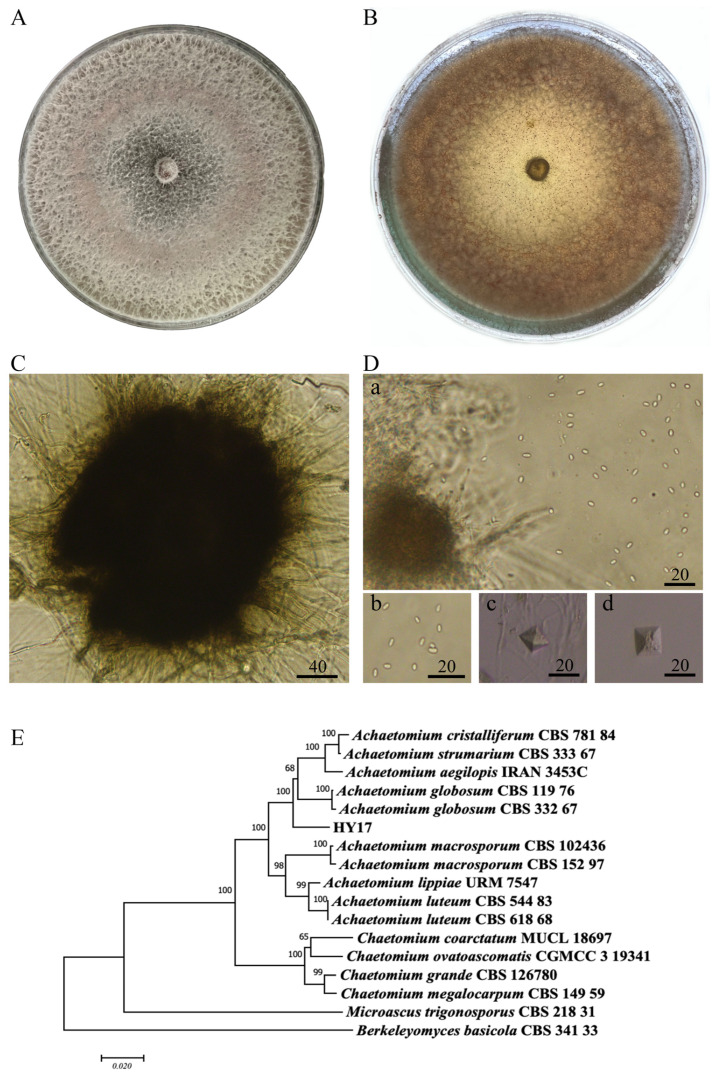
Morphological characteristics and molecular identification of the endophytic fungus HY17 isolated from *Sophora alopecuroides* based on *ITS-LSU-TUB* polygene tandem. (**A**,**B**) Colony of fungus HY17; (**C**) The microscopic morphology of ascus of fungus HY17; (**D**) Spores of fungus HY17 (a,b. ascospore, c,d. Chlamydospore; Scale unit: μm); (**E**) Fungus HY17 polygenic phylogenetic tree (*ITS-LSU-TUB*).

*Achaetomium* sp. HY17 filled the Petri dish with cotton-like colonies after being cultured on PDA medium at 28 °C under alternating light and dark conditions for 6 days (Figure 1A). The aerial mycelium was initially white and turned light pink after 6 days. The culture’s bottom was reddish-brown (Figure 1B). After 25 days of incubation at 28 °C, the ascus shell matured. It exhibited a dark brown or black color and could be either supergenic or buried in aerial hyphae. The ascus shell had pores and a spherical shape, with scattered, uneven, and irregularly branching accessory filaments covering its surface (Figure 1C). The asci were clubbed and transparent, containing oval-shaped ascospores (Figure 1(D(a),D(b))). Additionally, there were chlamydospores with a four-horned star shape under dark conditions (Figure 1(D(c),D(d))). These morphological characteristics bear some resemblance to those of the *Achaetomium fungal* species. Based on morphological identification, a phylogenetic tree was constructed using the tandem sequences of *ITS-LSU-TUB* multigenes (Figure 1E). The tree reveals that strain HY17 is closely related to *Achaetomium* sp., with these fungi clustering together with 100% support. However, strain HY17 is genetically distant from other strains whose species names have been determined, such as *Achaetomium cristalliferum* (CBS 781.84), *Achaetomium strumarium* (CBS 333.67), *Achaetomium aegilopis* (IRAN 3453C), and *Achaetomium globosum* (CBS 119.76), and forms an independent branch. Combining the morphological characteristics of strain HY17 and the alignment results based on the *ITS-LSU-TUB* multi-gene tandem sequence (Figure 1), strain HY17 was identified as a new species belonging to *Ascomycotina*, *Pyrenomycetes*, *Sordariales*, *Chaetomiaceae*, and *Achaetomium*. Since it was first isolated from *sophora alopecuroides*, it has been named *Achaetomium sophora* HY17. The *A. sophora* HY17 strain’s preservation number is CGMCC No.41060, and it has been deposited in the General Microbiology Center of the Chinese Committee for Microbial Culture Preservation. The percentage data of sequence difference (Supplementary Table S6) provides solid and reliable evidence that *A. sophora* HY17 is a new species.

### 3.3. Morphological Alterations of Co-Cultivated Fungal Mycelia

**Figure 2:**
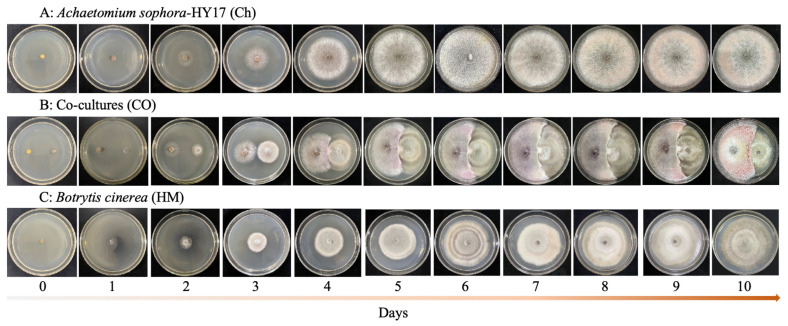
Morphological alterations of *A. sophora* HY17 co-cultured with *B. cinerea* HM1 for 0–10 days. (**A**) Monoculture of *A. scaphora* HY17 (Ch); (**B**) Co-cultures of *A. sophora* HY17 and *B. cinerea* HM1 (CO); (**C**) Monoculture of *B. cinerea* HM1 (HM).

The mycelial morphology of *A. sophora* HY17 and *B. cinerea* HM1 changed significantly during 0–10 days of co-culture compared to single cultures. Contact between *A. sophora* HY17 and *B. cinerea* HM1 was observed at 3 dpi, followed by obvious inhibition at 4 dpi with a light pink inhibition zone in the contact area. Over time, the color of the *A. sophora* HY17 colony and the contact area between the two fungi deepened significantly (Figure 2).

### 3.4. Quality Control of Metabolomic Samples and Comprehensive Sample Analysis

**Figure 3:**
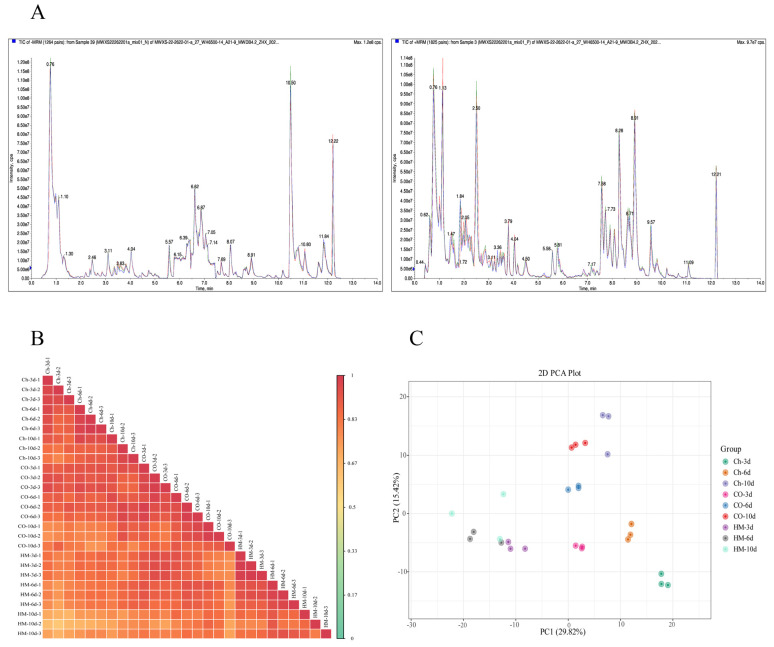
Detection and analysis of metabolomic-like essence-spectrum in population samples. (**A**) The essence spectrum of the QC sample was analyzed to detect the overlap map of total ion chromatogram (TIC) (The left is the negative ion mode, and the right is the positive ion mode, Different colors represent different time detection curves of the same sample by mass spectrometry.); (**B**) Repetitive evaluation of correlation in test samples; (**C**) Principal Component Analysis (PCA) score plot for the entire sample set. (n = 3, the same below).

UPLC-MS/MS was used to analyze three replicates of *A. sophora* HY17 and *B. cinerea* HM1 monocultures (Ch/HM) and co-cultures (CO) at 3, 6, and 10 dpi. The instrument’s high stability guaranteed the repeatability and reliability of the data. Overlapping display analysis of the total ion current maps (TIC graphs) for different quality control (QC) samples showed that metabolite detection in the negative ionization mode (NI) and positive ionization mode (PI) exhibited consistent retention time and peak intensity (Figure 3A). These results indicate the good signal stability of the mass spectrometry when detecting the same sample at different times. The proportion of substances with a CV value less than 0.5 in the QC samples is higher than 85%, indicating that the experimental data is stable, while the proportion of substances with a CV value less than 0.3 is higher than 75%, indicating that the experimental data is very stable (Supplementary Figure S2). The Pearson correlation coefficient |r| of the samples is close to 1, indicating strong correlation and high data reliability. Principal Component Analysis (PCA), a non-supervisory statistical method, can be used to analyze variables within a set of samples without known grouping and identify the principal components that best distinguish between samples (Figure 3B). By analyzing the sample score chart using PCA for the entire dataset (Figure 3C), it was found that PC1 represents 27.07% of the total variance, and PC2 represents 14.50%, which together account for 41.57% of the total variance. Clear separation trends between sample groups were observed, with good clustering of replicates within each group, indicating significant differences between samples and credible data processing results. In this study, the metabolite data of the samples were analyzed from multiple angles using univariate and multivariate statistical analyses to accurately mine differential metabolites. The PLS-DA and OPLS-DA models were established and verified through 200 permutations, obtaining nine comparison groups, and the values of R^2^ and Q^2^ were both high (Supplementary Figure S3). Differences in the metabolites obtained for different samples were significant, indicating that the model had good predictive ability and reliability to detect differential metabolites. In summary, through strict quality control and multivariate statistical analysis of UPLC-MS/MS data, the research ensured the high reliability of the data.

### 3.5. Dynamic Analysis of Differential Metabolites in A. sophora HY17 Monocultures

**Figure 4:**
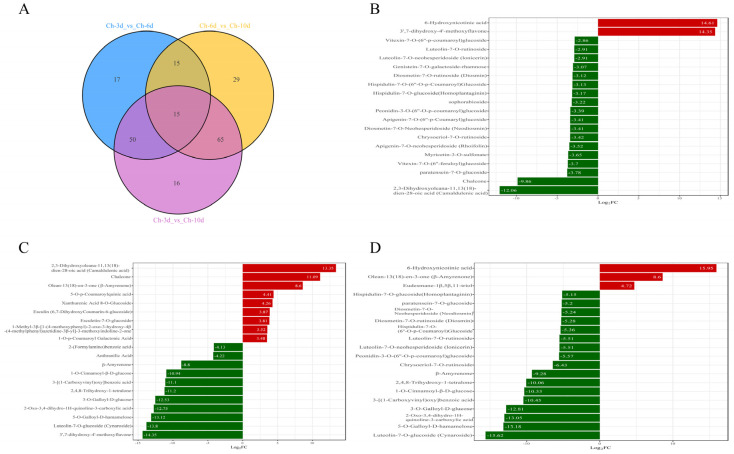
Dynamic alterations in metabolite profiles of *A. sophora* HY17 under isolated culture conditions. (**A**) Venn diagram illustrating the distinct metabolites of *A. sophora* HY17 cultured in isolation at various time points; (**B**–**D**) Comparisons of metabolite profiles between Ch-3d and Ch-6d (**B**), Ch-6d and Ch-10d (**C**), and Ch-3d and Ch-10d (**D**) were conducted.

To understand the changes in secondary metabolites synthesized by *A. sophora* HY17 during growth and development, we screened for differential metabolites using the VIP values (VIP > 1) obtained from a combined OPLS-DA model and the FC values (foldchange ≥ 2 and foldchange ≤ 0.5) obtained from univariate analysis as the criteria. We also compared the metabolites of *A. sophora* HY17 cultured alone at three time points using pairwise ensemble. It was found that *A. sophora* HY17 had the highest number of differential metabolites (146 species) when cultured alone at 3 dpi and 10 dpi. Fifteen differential metabolites were common across all three sets, while there were 17, 29, and 16 unique differential metabolites in the Ch-3d_vs_Ch-6d, Ch-6d_vs_Ch-10d, and Ch-3d_vs_Ch-10d sets, respectively (Figure 4A). In *A. sophora* HY17 monocultures, 6-Hydroxynicotinicacid and 3′,7-Dihydroxy-4′-methoxyflavone were significantly up-regulated in the early to middle stage (Figure 4B), while Camaldulenic acid was significantly down-regulated. Metabolites such as Chalcone were significantly up-regulated from the middle to late stage (Figure 4C), while Luteolin-7-*O*-glucoside (Cynaroside) and 5-*O*-Galloyl-D-hamamelose were significantly down-regulated. From the perspective of the whole growth cycle (Figure 4D), 6-Hydroxynicotinicacid showed the largest increase, with a fold change of 15.95. Luteolin-7-*O*-glucoside (Cynaroside) was the main down-regulated metabolite, with a fold change of −15.62. In conclusion, during the growth and development of *A. sophora* HY17, the key secondary metabolites include 6-Hydroxynicotinicacid, 3′,7-dihydroxy-4′-methoxyflavone, Camaldulenic acid, Chalcone, and Cynaroside, though most differential metabolites are down-regulated.

### 3.6. Metabolic Dynamics During the Interaction Between A. sophora HY17 and B. cinerea HM1

**Figure 5:**
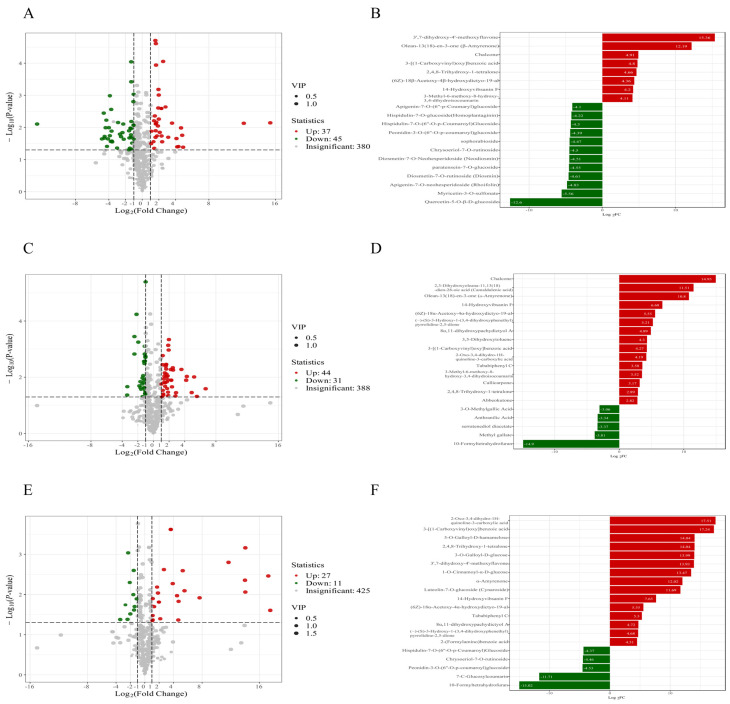
Comparison of differential metabolites between *A. sophora* HY17 monoculture and co-culture. (**A**,**C**,**E**) Volcanic heatmaps depicting differential metabolites of Ch-3d_vs_CO-3d (**A**), Ch-6d_vs_CO-6d (**C**), Ch-10d_vs_CO-10d (**E**); (**B**,**D**,**F**) Bar charts depicting the top 20 differential metabolites of Ch-3d_vs_CO-3d (**B**), Ch-6d_vs_CO-6d (**D**), and Ch-10d_vs_CO-10d (**F**). Each dot in the volcanic map represents a metabolite, in which the green dot represents the down-regulated differential metabolite, the red dot represents the up-regulated differential metabolite, and the gray color represents the detected metabolite with no significant difference; The abscissa represents the logarithmic value (log2FC) of the relative content difference multiple of a metabolite in two groups of samples. The greater the absolute value of the abscissa, the greater the relative content difference in the substance between two groups of samples. Under the screening conditions of VIP + FC + *p*-value, the ordinate indicates the level of difference significance (−log10 *p*-value), and the size of dots represents the VIP value. The abscissa of the bar chart of differential multiples is log2FC of the differential metabolites, that is, the differential multiples of the differential metabolites take the logarithm value based on 2, and the ordinate is the differential metabolites. Red indicates that the metabolite content is up-regulated, and green indicates that the metabolite content is down-regulated. Same as below.

Analysis of the dynamic changes in metabolites during the co-culture of *A. sophora* HY17 and *B. cinerea* HM1 showed that, compared with *A. sophora* HY17 monoculture, Ch-3d_vs_CO-3d exhibited significant up-regulation of 37 metabolites and significant down-regulation of 45 metabolites at 3 dpi (Figure 5A). Among the metabolites, flavonoids such as 3′,7-Dihydroxy-4′-methoxyflavone and Chalcone were significantly up-regulated, while Quercetin-5-*O*-β-D-glucoside and other substances were significantly down-regulated (Figure 5B). At 6 dpi of co-culture, Ch-6d_vs_CO-6d showed significant up-regulation of 44 metabolites and significant down-regulation of 31 metabolites (Figure 5C), with Chalcone and other substances being significantly up-regulated and 10-Formyltetrahydrofuran being significantly down-regulated (Figure 5D). At the end of the experiment on day 10, Ch-10d_vs _CO-10d showed twenty-seven significantly up-regulated metabolites and eleven significantly down-regulated metabolites (Figure 5E). Among these metabolites, 2, 4, 8-Trihydroxy-1-tetralone was found to be notably increased, while those that had been decreased included 10-Formyltetrahydrofuran and 7-C-Glucosylcoumarin (Figure 5F).

**Figure 6:**
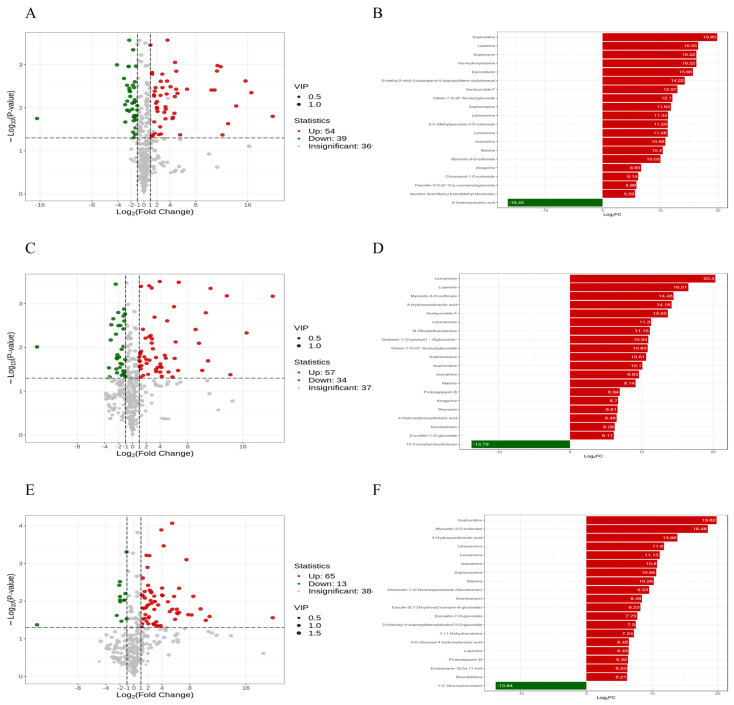
Comparison of differential metabolites between *B. cinerea* HM1 monoculture and co-culture. (**A**,**C**,**E**) Volcanic heatmaps depicting differential metabolites of HM-3d_vs_CO-3d (**A**), HM-6d_vs_CO-6d (**C**), HM-10d_vs_CO-10d (**E**); (**B**,**D**,**F**) Bar charts depicting the top 20 differential metabolites of HM-3d_vs_CO-3d (**B**), HM-6d_vs_CO-6d (**D**), and HM-10d_vs_CO-10d (**F**).

The control group consisted of *B. cinerea* HM1 culture alone. At 3 dpi, HM-3d_vs_CO-3d (Figure 6A) exhibited significant up-regulation of 54 metabolites and down-regulation of 39 metabolites. Notably, Sophoridine, Lupanine, and 14α-Hydroxymatrine were significantly up-regulated, while 6-Hydroxynicotinic acid was significantly down-regulated (Figure 6B). At 6 dpi, HM-6d_vs_CO-6d (Figure 6C) showed significant up-regulation of 57 metabolites and down-regulation of 34 metabolites. Among them, Lemannine, Lupanine, Lehmannine, Sophocarpine, and Sophoridine were significantly up-regulated; whereas 10-Formyltetrahydrofuran was significantly down-regulated (Figure 6D). At 10 dpi, analysis of the HM-10d_vs_CO-10d (Figure 6E) set revealed significant up-regulation of 65 metabolites and down-regulation of 13 metabolites. Notably, Sophoridine, Lehmannine, Lemannine, Isomatrine, Sophocarpine, Matrine, and other substances were significantly up-regulated, whereas 7-C-Glucosylcoumarin was significantly down-regulated (Figure 6F). Interestingly, when *B. cinerea* HM1 monoculture was used as the control, it was observed that alkaloids were continuously and significantly up-regulated during the co-culture process, which preliminarily indicated that alkaloids were rarely or even not produced during the culture process of *Botrytis cinerea* HM1. However, there was no significant difference between the monoculture and co-culture of *A. sophora* HY17. Therefore, it is inferred that alkaloids are produced by *A. sophora* HY17 during its growth and can be considered potential antifungal substances.

### 3.7. Quantitative Analysis of Differential Metabolites

**Figure 7:**
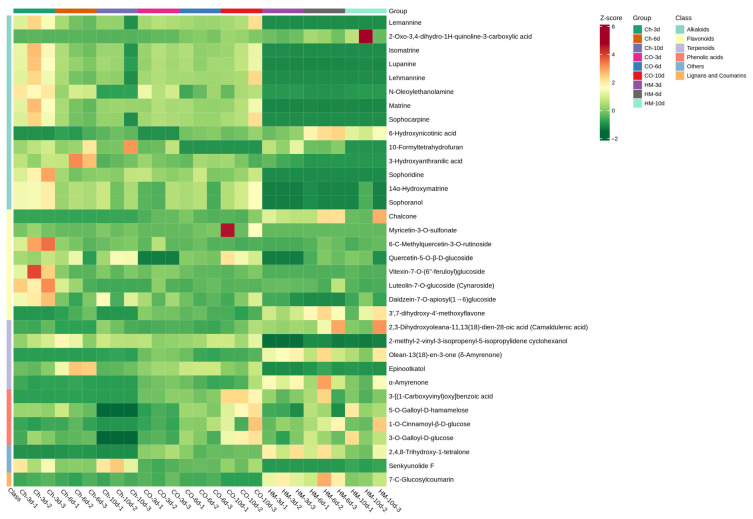
Heat map illustrating the results of relative quantitative analysis for differential metabolites (Log2FC > 10).

To determine the relative quantitative changes in differential secondary metabolites in each category, metabolites with Log2FC > 10 from the co-culture screening were selected for the quantitative analysis. The screened substances included 14 alkaloids, 8 flavonoids, 5 terpenoids, 4 phenolic acids, 1 lignan and coumarin, and 2 other classes. A cluster heat map (Figure 7) was generated.

The analysis of differential alkaloid metabolites revealed that Lemannine, Isomatrine, Lupanine, Lehmannine, Matrine, Sophocarpine, Sophoridine, 14α-Hydroxymatrine, and Sophoranol exhibited high levels in the monoculture of *A. sophora* HY17, particularly on the third day. Their levels gradually decreased with prolonged culture time but showed an increasing trend in the late co-culture stage. Conversely, the contents of 10-Formyltetrahydrofuran and 3-Hydroxyanthranilic acid were initially high in the *A. sophora* HY17 monoculture but displayed a decreasing tendency in the late co-culture stage. Furthermore, while the content of 2-OXo-3,4-dihydro-1H-quinoline-3-carboxylic acid was low under *A. sophora* HY17 monoculture conditions, it increased over time during co-cultivation. Additionally, a higher content of 6-Hydroxynicotinic acid was observed in the *B. cinerea* HM1 monoculture stage, which subsequently decreased during co-cultivation. The metabolism of flavonoids exhibited significant differences, with the levels of Chalcone and 3′,7-Dihydroxy-4′-methoxyflavone showing a substantial increase during co-culture compared to the monoculture of *A. sophora* HY17. Furthermore, on the 10th day of co-culture, there was a significant elevation in the content of Luteolin-7-*O*-glucoside (Cynaroside) when compared with the *A. sophora* HY17 monoculture; in contrast, on the 3rd day of co-culture, there was a notable decrease in Quercetin-5-*O*-β-D-glucoside content relative to the *A. sophora* HY17 monoculture. Additionally, Myricetin-3-*O*-sulfonate, 6-C-methylQuercetin-3-*O*-Rutinoside, Vitexin-7-*O*-(6′-Feruloyl) glucoside, and Daidzein-7-*O*-apiosyl (1→6) glucoside were found to have higher concentrations in the *A. sophora* HY17 monoculture, but these metabolites experienced a significant increase during co-cultivation when compared with the *B. cinerea* HM1 monoculture.

The terpenoids 2,3-Dihydroxyoleana-11,13(18)-dien-28-oic acid (Camaldulenic acid) and Olean-13(18)-en-3-one were found to have lower levels in the monoculture of *A. sophora* HY17 but higher levels in the monoculture of *B. cinerea* HM1. However, their contents significantly increased compared with that of the *A. sophora* HY17 monoculture after co-culture. The contents of Epinootkatol and 2-Methyl-2-vinyl-3-isopropenyl-5-isopropylidene cyclohexanol were observed to be higher in the monoculture of *A. sophora* HY17 and showed a significant increase during co-culture when compared with the *B. cinerea* HM1 monoculture. The levels of phenolic acids, including 3-[(1-Carboxyvinyl)oxy]benzoic acid, 5-*O*-Galloyl-D-hamamelose, 1-*O*-Cinnamoyl-β-D-glucose, and 3-*O*-Galloyl-D-glucose, were found to have increased after co-culture compared with *A. sophora* HY17 monoculture. Among these phenolic acids, Cinnamoyl-β-D-glucose exhibited the highest levels on the 10th day of co-culture. Furthermore, the content of 7-C-Glucosylcoumarin was higher in the *B. cinerea* HM1 monoculture but decreased during co-culture. The content of 2,4,8-trihydroxy-1-tetralone was significantly up-regulated during co-culture compared with the *A. sophora* HY17 monoculture but still lower than that of the *B. cinerea* HM1 monoculture. Additionally, the content of Senkyunolide F decreased during co-culture compared with the *A. sophora* HY17 monoculture; however, it showed an obvious trend towards up-regulation when compared with the *B. cinerea* HM1 monoculture.

### 3.8. Enrichment Analysis of Differential Metabolites Performed Using the KEGG Database

**Figure 8:**
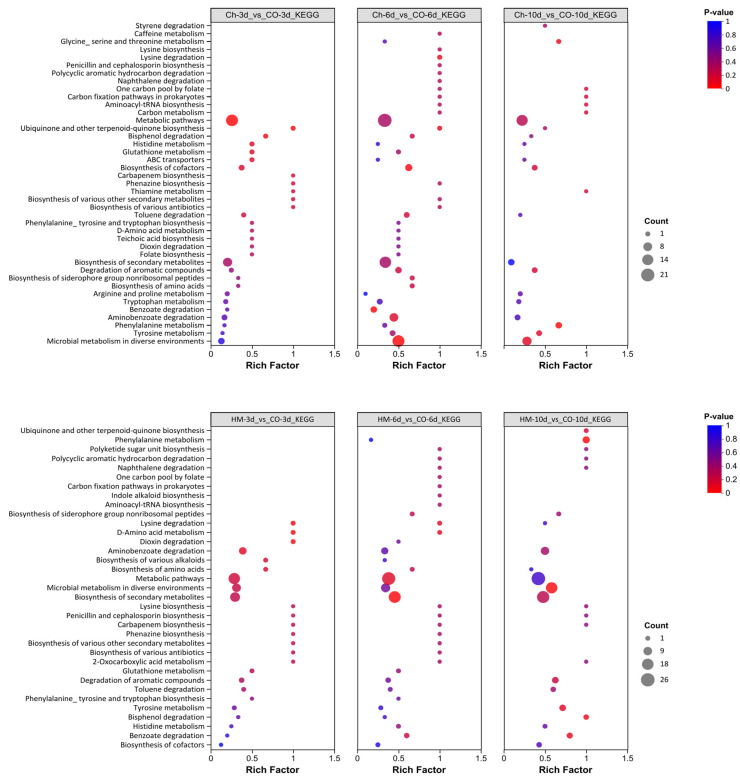
KEGG classification and enrichment analysis of differential metabolites. The horizontal coordinate represents the Rich Factor corresponding to each pathway, while the vertical coordinate denotes the path name (sorted by *p*-value). The color of the dots reflects the magnitude of the *p*-value, with red indicating a higher level of significance in enrichment. Additionally, the size of the dots indicates the extent to which differential metabolites are enriched.

To gain insights into the underlying pathways associated with differential metabolites, we performed enrichment analysis on the KEGG annotation results obtained for metabolites exhibiting significant differences (Figure 8). The metabolic pathways that were most significantly enriched in the co-cultured samples at 3 dpi compared with the control samples included ubiquinone and other terpenoid–quinone biosynthesis, benzoate degradation, ABC transporters, glutathione metabolism, histidine metabolism, and biosynthesis of cofactors. Additionally, D-Amino acid metabolism emerged as the most significantly enriched pathway in the HM-3d_vs_CO-3d comparison. This comparison also revealed enrichment in dioxin degradation, lysine degradation, aminobenzoate degradation, biosynthesis of amino acids and various alkaloids, metabolic pathways, microbial metabolism in diverse environments, and biosynthesis of secondary metabolites. The most significantly enriched metabolic pathway in the Ch-6d_vs_CO-6d co-culture at 6 dpi was identified as microbial metabolism in diverse environments. Additionally, analysis of this co-culture revealed involvement in various biosynthetic processes such as benzoate degradation, cofactor biosynthesis, lysine degradation, ubiquinone and other terpenoid–quinone biosynthesis, aminobenzoate degradation, toluene degradation, and aromatic compound degradation. In contrast, the key pathway enriched in HM-6d_vs_CO-6d was biosynthesis of secondary metabolites. Furthermore, D-amino acid metabolism and lysine degradation pathways were also observed, along with metabolic pathways related to benzoate degradation and amino acid biosynthesis including Siderophore nonribosomal peptides. The primary metabolic pathway enriched in the metabolite set of Ch-10d_vs_CO-10d at 10 dpi was phenylalanine metabolism. Other enriched pathways were glycine, serine, and threonine metabolism; microbial metabolism in diverse environments; tyrosine metabolism; biosynthesis of cofactors; and degradation of aromatic compounds. The most significantly enriched pathway in the HM-10d_vs_CO-10d set was phenylalanine metabolism. Additional enriched pathways included microbial metabolism in diverse environments, bisphenol degradation, benzoate degradation, tyrosine metabolism, ubiquinone and other terpenoid–quinone biosynthesis, degradation of aromatic compounds, and biosynthesis of terpenoid–quinone secondary metabolites.

Overall, the biosynthetic pathways of secondary metabolites were consistently and significantly enriched in the co-cultures relative to the monocultures, regardless of whether the monocultures were cultured with strain HY17 alone or *B. cinerea* HM1 cinesulfum alone. There was significant enrichment in phenylalanine metabolism at 6 and 10 dpi.

### 3.9. Verification of Antifungal Activity of Different Metabolites and Determination of Their Sources

**Figure 9:**
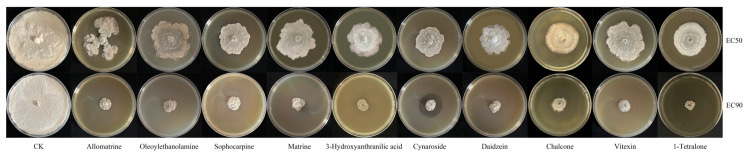
Inhibition of *B.cinerea* HM1 by active metabolites.

The inhibitory effect of the screened differential metabolites on *B. cinerea* HM1 growth was verified by conducting an antifungal test using a PDA medium containing 10 standard substances. Among these compounds, Sophoridine, Sophocarpine, Matrine, Oleoylethanolamine, 3-Hydroxy-2-aminobranilic acid, Chalcone, Luteolin, Daidzein, Vitexin, and 1-Tetrahydronaphthone exhibited varying degrees of inhibition against *B. cinerea* HM1. The respective virulence regression equations were used to calculate the EC_50_ and EC_90_ values for different fungistatic active metabolites (Table 2, Figure 9). Chalcone demonstrated the lowest inhibitory concentration among these compounds, with EC_50_ and EC_90_ values of 60.03 and 118.16 μg/mL, respectively.

The results of high-performance liquid chromatography (Supplementary Figure S5) showed that in the detection of alkaloids, the peaks of Matrine and Sophoridine as standard substances appeared at 9.338 min and 12.431 min, respectively, while these peaks appeared in the hyphal extract of *A. sophora* HY17 at 9.466 min and 12.506 min, respectively, which were similar to the chromatographic retention time of these alkaloid standard substances and exhibited good separation from other impurity peaks around them. In the detection of flavonoids, the peaks of Vitexin, Luteolin, Daidzein, and Chalcone appeared at 8.973, 9.622, 13.836, and 33.532 min, respectively, and the retention time of the *A. sophora* HY17 mycelium extract with flavonoid as the standard was 8.960, 9.640, 13.880, and 34.700 min, respectively. In addition, the results of mass spectrometry also showed that the above substances were produced and accumulated (Supplementary Figure S1 and Supplementary Table S1). Therefore, it is determined that *A. sophora* HY17 produces Matrine, Sophocarpine, Sophoridine, Vitexin, Luteolin, Daidzein and Chalcone, and all of these substances have antifungal activity.

## 4. Discussion

Endophytic fungi in medicinal plants exhibit diverse and valuable biological activities, serving as a significant source of bioactive natural products with potential applications in medicine, food, and agriculture [29]. A novel endophytic fungus, *Achaetomium sophora* HY17, was isolated from the medicinal plant *Sophora alopecuroides*. Through morphological and molecular biology analyses, it was identified as a new species. This is consistent with the results of previous studies [30]. It has been found that *Achaetomium fungi* are relatively new and rare endophytic fungi in most medicinal plants. To date, there are few reports on the metabolites and antifungal substances of *Achaetomium fungi*. In this study, *A. sophora* HY17 exhibited broad-spectrum antifungal activity against eight pathogenic fungi, including *B. cinerea* HM1. Compared to the simultaneous inoculation treatment, pre-inoculation of *A. sophora* HY17 three days before inoculation with pathogenic fungi significantly enhanced its inhibitory effect. *B. cinerea* HM1, *R. solani* pn5-2, *F. avenacea* YM1, and *C. siamense* NX2-7 were all inhibited by more than 90%. Plant endophytic fungi mainly inhibit the infection and proliferation of pathogenic fungi through competition, antibiosis, and hyperparasitism [31]. Pre-inoculated endophytic fungi occupy ecological sites, compete for nutrients, and produce antifungal that effectively inhibit the growth of pathogenic fungi, thus showing a good biocontrol effect [32]. As an endophytic fungus with environmental stability and strong colonization ability, *A. sophora* HY17 can rapidly compete for nutrients and space while producing antifungal substances that play a role in biocontrol.

In this study, the *A. sophora* HY17 strain, as a biocontrol fungus, was co-cultured with *B. cinerea* HM1 as an external stimulus to induce the synthesis and accumulation of secondary metabolites. Using UPLC-MS/MS metabolomic technology, we systematically explored the metabolic profile of *A. sophora* HY17 and identified alkaloids, flavonoids, phenolic acids, terpenes, lignans, and coumarin as the main metabolic substances, similar to the effective medicinal components identified in its host *Sophora alopecuroides* [33]. After screening, several differential metabolites were identified, with alkaloids (14 types) and flavonoids (8 types) being the most abundant and showing significant relative quantitative changes. Antifungal activity verification tests showed that alkaloids such as Sophoridine, Sophocarpine, Matrine, Oleoylethanolamine, and 3-Hydroxyanthranilic acid, as well as flavonoids like Chalcone, Cynaroside, Daidzein, and Vitexin, significantly inhibited the growth of *B. cinerea*. HPLC analysis of mycelial extracts showed that these differential metabolites were potential antifungal substances produced by *A. sophora* HY17.

Alkaloids are secondary metabolites produced by living organisms during evolution to adapt to the natural environment and act as natural phytoalexins. Sun et al. [34] discovered that Matrine, Oxymatrine, Sophocarpine, and Oxysophocarpine exhibited antimicrobial effects against *Staphylococcus aureus*, *Escherichia coli*, *Pseudomonas aeruginosa*, *beta-hemolytic Streptococcus*, and *Candida albicans*. In this study, alkaloid metabolites such as Sophoridine, Sophocarpine, Matrine, Oleoylethanolamine, and 3-Hydroxyanthranilic acid demonstrated potent antifungal activity. Comparative analysis revealed significant enrichment of alkaloid biosynthetic pathways during the interaction between *A. sophora* HY17 and *B. cinerea* HM1, with several alkaloids being significantly up-regulated in the co-culture compared with the *B. cinerea* HM1 monoculture. Among the up-regulated quinolone alkaloids were Matrine, Sophoridine, and Sophocarpine. The effective alkaloid composition was consistent with that reported in the host plant *Sophora alopecuroides*, with both displaying broad-spectrum antifungal activity [35]. In the 1960s, researchers from the former Soviet Union isolated and extracted 18 flavonoids from licorice [36]. These flavonoids exhibited antifungal activity against certain Candida species, including Candida parapsilosis, Candida orthopsilosis, and Candida metapsilosis [37]. Eight differential metabolites of flavonoids were detected in the co-culture samples *A. sophora* HY17 and *B. cinerea* HM1. Among them, Chalcone, 3′,7-dihydroxy-4′-methoxyflavone, Quercetin-5-*O*-β-D-glucoside, and Luteolin-7-*O*-glucoside showed a significant increase in content during co-culture compared with the *A. sophora* HY17 monoculture. The most significant change was observed for Chalcone, which exhibited a notable up-regulation trend at 3 dpi and extremely high up-regulation at 6 dpi.

Additionally, the presence of terpenoids, phenolic acids, and coumarin compounds was detected in the co-culture samples of *A. sophora* HY17 and *B. cinerea* HM1. Previous studies have reported the isolation of 516 terpenoids from various plant endophytic fungi, which primarily have cytotoxic, antifungal, and anti-inflammatory activities [38]. Phenolic acids are significant natural secondary metabolites with considerable research value and application potential [39]. Studies have revealed that phenolic acids extracted from plum fruits display inhibitory activities against six pathogenic microorganisms: *Xanthomonas vesicatoria*, *Botryosphaeria dothidea*, *Colletotrichum sisamense*, *Phytophthora capsici*, *Fusarium solani*, and *Fusarium graminearum* [40]. Coumarin compounds are widely present in medicinal plants and possess various biological activities, including information transmission, insecticidal, anti-cancer, and antifungal properties [41]. In addition to these compounds, this study also detected two other differential metabolites: 2,4,8-Trihydroxy-1-tetralone and Ligusticolactone F, a type of phthalide that can be extracted from medicinal plants like *Angelica sinensis* and *Ligusticum chuanxiong*. Pharmacological studies have demonstrated the wide range of pharmacological effects exhibited by Ligusticolide compounds, such as antioxidative effect, anti-inflammatory activity, and anticoagulation property [42]. Furthermore, this study found that 1-tetralone exhibited potent inhibitory effect against *B. cinerea* HM1.

The phenylalanine metabolic pathway is crucial for the synthesis of various bioactive metabolites. In this pathway, the key substrate is phenylalanine, which is catalyzed by key enzymes such as phenylalanine ammonia-lyase (PAL), cinnamic acid 4-hydroxylase (C4H), and 4-coumaric acid coenzyme A ligase (4CL). The reaction produces intermediates such as coumaric acid, sinapic acid, trans-cinnamic acid, and ferulic acid. These intermediates are then transformed into chlorogenic acid, coumarin, trans-coumaryl-coa, and other compounds. Through multiple reaction pathways [43,44,45], these metabolites further generate secondary metabolites with pharmacological activities, including flavonoids, alkaloids, lignin, and benzoate glycosides, which play a vital role in fungal growth, development, and adaptation to the environment. Lu et al. [46] sequenced the genome of the endophytic vine fungus *Alternaria* MG1 and identified the phenylalanine metabolic pathway as playing a crucial role in secondary metabolite synthesis. Twenty-one proteins encoded by multiple genes were found to catalyze/support the biosynthesis of flavonoids, alkaloids, and lignin in this pathway. In this study, KEGG enrichment analysis of differential metabolites revealed significant enrichment of coumarins, flavonoids, and alkaloids in the phenylalanine metabolic pathway during the co-culture of *A. sophora* HY17 with *B. cinerea* HM1 in the middle and late stages. Similarly, Chen et al. [47] found that *Streptomyces virginiae* XDS1-5 produced differential metabolites with antibacterial activity that were enriched in the phenylalanine metabolic pathway, such as indoleines. These findings suggest that the phenylalanine metabolic pathway may be important for synthesizing secondary metabolites like flavonoids and alkaloids during the interaction between *A. sophora* HY17 and *B. cinerea* HM1.

There are some limitations to the current study. Even though the current study identified *A. sophora* HY17 as a major potential antifungal species, its metabolic characteristics and antifungal mechanisms remain largely unknown. In the next step, the antifungal substances produced by *A. sophora* HY17 will be identified through separation and extraction. In addition, we will examine the phenylalanine metabolic pathway and utilize whole-genome and comparative transcriptomic analyses to identify key genes involved in alkaloid and flavonoid synthesis in order to elucidate the complete biosynthetic pathway of these compounds in *A. sophora* HY17. Overall, the current study provides a theoretical foundation for enhancing the production of antifungal substances in *A. sophora* HY17 through genetic modification. To sum up, our research reveals the strong antagonistic ability of HY17 against many plant pathogens and the related mechanisms. These findings confirm the great potential of HY17 as an efficient and environmentally friendly biological control agent. Developing it into a commercial microbial inoculum will provide a new and promising option for reducing the use of chemical pesticides, coping with the drug resistance of pathogenic bacteria, and developing green and sustainable agriculture, which has important theoretical and practical significance.

## 5. Conclusions

*A. sophora* HY17 exhibits broad-spectrum antifungal activity and possesses significant biocontrol potential as a resource fungus. Based on the co-culture approach, UPLC-MS/MS metabolomic analysis preliminarily identified alkaloids and flavonoids as the main active substances produced during the interaction between *A. sophora* HY17 and *B. cinerea* HM1. Ten differential metabolites, including Sophoridine, Sophocarpine, Matrine, Oleoylethanolamine, 3-Hydroxyanthranilic acid, Chalcone, Cynaroside, Daidzein, Vitexin, and 1-Tetralone, were detected. These metabolites are primarily involved in the phenylalanine metabolic pathways. This study demonstrates that *A. sophora* HY17 is a natural producer of antifungal substances with important potential.

## Figures and Tables

**Table 1:** Inhibition rate of endophytic fungus HY17 against eight strains of pathogenic fungi.

Pathogenic Fungi	Inhibition Rate/%	
	Simultaneous Inoculation	Delivery of HY17 for 3 Days Prior
*Botrytis cinerea* HM1	74.60 ± 2.75 ^a^	92.86 ± 2.38 ^a^
*Rhizoctonia solani* pn5-2	62.81 ± 1.34 ^a^	99.05 ± 0 ^a^
*Fusarium avenacea* YM1	54.18 ± 2.41 ^b^	96.30 ± 1.50 ^a^
*Fusarium oxysporum* pm29-3	49.49 ± 2.85 ^bc^	86.87 ± 2.86 ^c^
*Colletotrichum siamense* NX2-7	68.08 ± 2.09 ^a^	92.44 ± 1.26 ^b^
*Fusarium tricuspidata* pm36-8	49.52 ± 3.39 ^bc^	84.84 ± 0 ^c^
*Clonostachys rosea* pm34-5	53.22 ± 2.49 ^bc^	54.20 ± 2.64 ^d^
*Pythium aphanidermatum* pn8-3	46.42 ± 1.56 ^c^	86.12 ± 1.35 ^c^

Data in the table represent the mean ± standard error for each treatment with three replicates. Different lowercase letters indicate that the inhibition rate of strains is significantly different at the *p* < 0.05 level (n = 3).

**Table 2:** Regression equations for the toxicity of ten active metabolites inhibiting *B.cinerea* HM1.

Compounds	Virulence Regression Equation	R^2^	EC_50_ (μg/mL)	EC_90_ (μg/mL)	95% Confidence Intervals
Sophoridine	Y = 0.4902 × X − 44.04	0.9801	191.84	273.44	0.4483–0.5321
Oleoylethanolamine	Y = 0.3554 × X − 21.80	0.9868	202.02	314.58	0.3336–0.3772
Sophocarpine	Y = 0.3599 × X − 20.76	0.9902	196.61	307.75	0.3409–0.3789
Matrine	Y = 0.3695 × X − 16.12	0.9791	178.94	287.21	0.3409–0.3981
3-Hydroxyanthranilic acid	Y = 0.4791 × X − 43.43	0.9685	195.01	278.51	0.4273–0.5308
Cynaroside	Y = 0.4419 × X − 37.04	0.9835	196.97	287.48	0.4015–0.4823
Daidzein	Y = 0.3366 × X − 21.12	0.9692	211.29	330.12	0.3048–0.3684
Chalcone	Y = 0.6828 × X + 9.011	0.9854	60.03	118.61	0.6387–0.7269
Vitexin	Y = 0.3415 × X − 22.00	0.9675	210.83	327.96	0.2974–0.3856
1-Tetralone	Y = 0.2473 × X + 34.63	0.9894	62.15	223.91	0.2244–0.2702

## Data Availability

The original contributions presented in this study are included in the article/Supplementary Material. Further inquiries can be directed to the corresponding author.

## Supplementary Materials

The following supporting information can be downloaded at: https://www.mdpi.com/article/10.3390/microorganisms13122794/s1, Table S1: Statistical table of antifungal substance mass spectrometry results. Table S2: Source and identification information of pathogenic fungi. Table S3: Q1 and Q3 for representative metabolite. Table S4: Software List information. Table S5: Sequences for the molecular identification of HY17. Table S6: Sequence difference analysis. Figure S1: Antifungal substance mass spectrometry results. (A) Negative ion mode; (B) Positive ion mode. Figure S2: CV distribution diagram of each group of samples. The abscissa represents the CV value, and the ordinate rep-resents the ratio of the number of substances less than the corresponding CV value to the total number of substances. Different colors represent different grouped samples, and QC is the quality control sample. The CV values corresponding to the two reference lines perpendicular to the X axis are 0.3 and 0.5, and the number of substances corresponding to the two reference lines parallel to the X axis accounts for 75% and 85% of the total number of substances. Figure S3: OPLS-DA permutation. The abscissa indicates the R2Y and Q2 values of the model, and the ordinate indicates the frequency of the model classification effect in 200 random permutation and combination experiments. In the figure, orange represents the random grouping model R2Y, purple represents the random grouping model Q2, and the values represented by the black arrows are R2X, R2Y and Q2 of the original model. (From left to right, it is Ch-3d vs HM-3d vs CO-3d, Ch-6d vs HM-6d vs CO-6d and Ch-10d vs HM-10d vs CO-10d.). Figure S4: Integral correction chart for quantitative analysis of metabolites. Above is positive ion mode; The following is negative ion mode. The abscissa is the retention time (min) of metabolite detection, and the ordinate is the ion current intensity (cps) of a metabolite ion detection. Figure S5: High-performance liquid chromatograms of three alkaloid and four flavonoid standards and mycelium extracts of A. sophora HY17. (A) Three alkaloid standards (1: Matrine; 2: Sophocarpine and Sophoridine); (B) Detection of alkaloids in hyphal extracts of A. sophora HY17; (C) four flavonoid standards (1: Vitexin; 2: Luteolin; 3: Daidzein; 4: Chalcone); (D) Fla-vonoid detection of hyphal extracts from A. sophora HY17.
