# Supplementary material for: Metabolomic Analysis of Antifungal Secondary Metabolites from Achaetomium sophora HY17 in Co-Culture with Botrytis cinerea HM1
Source: Microorganisms. 2025 Dec 8;13(12):2794. doi: 10.3390/microorganisms13122794 (PMC12736334; doi:10.3390/microorganisms13122794)
Supplement: Supplementary file 1 [file microorganisms-13-02794-s001.zip › microorganisms-3954286-supplementary.pdf]

Table S1. Statistical table of antifungal substance mass spectrometry results

Index	Compounds	Class I	Ch-3d-1	Ch-3d-2	Ch-3d-3	Ch-6d-1	Ch-6d-2	Ch-6d-3	Ch-10d-1	Ch-10d-2	Ch-10d-3
mws1101	Matrine	Alkaloids	2.73E+07	4.49E+07	3.24E+07	1.83E+07	1.19E+07	9.20E+06	1.61E+07	1.67E+07	1.60E+07
mws1100	Sophocarpine	Alkaloids	2.72E+07	4.44E+07	3.22E+07	1.88E+07	1.17E+07	1.01E+07	1.61E+07	1.57E+07	9.00E+00
mws1108	Sophoridine	Alkaloids	1.26E+07	1.72E+07	2.38E+07	8.48E+06	5.45E+06	6.65E+06	7.24E+06	6.62E+06	1.05E+07
mws0983	N-Oleoylethanolamine	Alkaloids	8.07E+04	7.20E+04	8.03E+04	3.22E+04	5.17E+04	4.96E+04	5.07E+03	5.40E+03	5.80E+03
mws0596	3-Hydroxyanthranilic acid	Alkaloids	2.55E+05	1.77E+05	2.72E+05	2.66E+05	6.77E+05	5.80E+05	7.12E+04	9.68E+04	1.21E+05
mws1731	Chalcone	Flavonoids	1.20E+04	5.04E+03	8.00E+03	9.00E+00	9.00E+00	9.00E+00	1.36E+04	2.02E+04	2.51E+04
pmb0678	Vitexin-7-O-(6''-feruloyl)glucoside	Flavonoids	1.49E+05	3.87E+05	2.55E+05	4.76E+04	7.13E+03	6.02E+03	4.22E+04	1.10E+04	4.09E+04
pme2459	Luteolin-7-O-glucoside (Cynaroside)	Flavonoids	4.46E+05	3.61E+05	5.56E+05	2.35E+05	1.35E+05	1.46E+04	9.00E+00	9.00E+00	9.00E+00
Zmdp003228	Daidzein-7-O-apiosyl(1→6)glucoside*	Flavonoids	6.22E+04	7.75E+04	9.41E+04	2.48E+04	9.16E+03	2.57E+04	7.01E+04	1.30E+04	5.51E+04
Wchn004836	2,4,8-Trihydroxy-1-tetralone	Others	1.22E+04	9.85E+03	6.90E+03	1.41E+04	2.78E+04	2.15E+04	9.00E+00	9.00E+00	9.00E+00

Table S2. Source and identification information of pathogenic fungi.

Pathogenic fungi	Source of isolation	Sample collection location	Methods of identification
Botrytis cinerea HM1	grape fruit infected with botrytis cinerea	Chateau Hedong, Ningxia	phenotypic diversity division<br>*Bc-hch* sequence amplification<br>*Flipper* and *Boty* transposon detection.
Rhizoctonia solani pn5-2	alfalfa root rot samples	Yinchuan, Ningxia	phenotypic diversity division,<br>*rDNA-ITS* gene sequence analysis<br>*β-tubulin* gene sequence analysis.
Fusarium avenacea YM1	alfalfa root rot samples	Yinchuan, Ningxia	
Fusarium oxysporum pm29-3	alfalfa root rot samples	Yinchuan, Ningxia	
Colletotrichum siamense NX2-7	alfalfa root rot samples	Yinchuan, Ningxia	
Fusarium tricuspidata pm36-8	alfalfa root rot samples	Yinchuan, Ningxia	
Clonostachys rosea pm34-5	alfalfa root rot samples	Yinchuan, Ningxia	
Pythium aphanidermatum pn8-3	alfalfa root rot samples	Yinchuan, Ningxia	

Table S3. Q1 and Q3 for representative metabolite.

Index	Compounds	Q1 (Da)	Q3 (Da)
mws1101	Matrine	2.49E+02	1.48E+02
mws1100	Sophocarpine	2.47E+02	1.36E+02
mws1108	Sophoridine	2.49E+02	1.50E+02
mws0983	N-Oleoylethanolamine	3.26E+02	6.21E+01
mws0596	3-Hydroxyanthranilic acid	1.54E+02	1.36E+02
mws1731	Chalcone	2.09E+02	7.70E+01
pmb0678	Vitexin-7-O-(6''-feruloyl)glucoside	7.71E+02	1.77E+02
pme2459	Luteolin-7-O-glucoside (Cynaroside)	4.49E+02	2.87E+02
Zmdp003228	Daidzein-7-O-apiosyl(1→6)glucoside*	5.49E+02	2.55E+02
Wchn004836	2,4,8-Trihydroxy-1-tetralone	1.93E+02	1.75E+02

Table S4. Software List information.

**Analysis**	**Software**	**Version**	**Data processing methods**
PCA	R (base package)	3.5.1	UV（unit variance scaling）
Heat map	R (ComplexHeatmap)	2.8.0	UV（unit variance scaling）
Pearson's correlation coefficient	R (base package; Hmisc)	3.5.1; 4.4.0	-
Correlation plots between samples	R (corrplot)	0.84	-
OPLS-DA	R (MetaboAnalystR)	1.0.1	log2 conversion+centralization

Table S5. Sequences for the molecular identification of HY17.

Gene	Sequence
ITS	TACCTACAACCGTTGCTTCGGCGGGCGGGCGCCCCGCGCGCCCCCCGGGCCCCCAC-<br>TCGCTGGGGGCGCCCGCCGGAGGTACCTAACTCTTGTTGATCACACGGCCTCTCTGAGTCTTCTGTACTG<br>AATAAGTCAAAACTTTCAACAACGGATCTCTTGGTTCTGGCATCGATGAAGAAC-<br>GCAGCGAAATGCGATAAGTAATGTGAATTGCAGAATTCAGTGAATCATCGAATCTTTGAACGCACATTG<br>CGCCCGCCAGTATTCTGGCGGGCATGCCTGTTCGAGCGTCATTTCAACCATCAA-<br>GCCCCCGGGCCTGTGTTGGGGACCTGCGGCTGCCCGCAGGCCCCGAAAACCAGTGGCGGGCTCGCTGTC<br>ACCCCGAGCGTAGTAGCTAACACCTCGCCCAGGGAGTGCCGCGGGTCCCGGCCGTAAAAC-<br>CTAACACCAAAGGTTGACCTCGGATCAGGTAGGAAGACCCGCTGAACTTAAGCATATCAA
LSU	TAGTAACGGCGAGTGAAGCGGCAACAGCTCAAATTTGAAATCTGGCTTCGGCCCGAGTT-<br>GTAATTTGTAGAGGAAGCTTTAGGCGCGGCACCTTCTGAGTCCCCTGGAACGGGGCGCCACAGAGGGT<br>GAGAGCCCCGTATAGTTGGATGCCTAGCCTGTGTAAAGCTCCTTCGACGAGTCGAGTAGTTT-<br>GGGAATGCTGCTCAAAATGGGAGGTAAATTTCTTCTAAAGCTAAATACCGGCCAGAGACCGATAGCGC<br>ACAAGTAGAGTGATCGAAAGATGAAAAGCACTTTGAAAAGAGGGTTAAATAGCAC-<br>GTGAAATTGTTGAAAGGGAAGCGCTTGTGACCAGACTTGCGCCGGGCGGATCATCCGGTGTTCTCACCG<br>GTGCACTCCGCCCGGCTCAGGCCAGCATCGGTTCTCGCGGGGGGATAAAGGCCACGGGAAC-<br>GTAGCTCCCCACGGGGAGTGTTATAGCCCGGGGCGCAATGCCCTCGCGGGGACCGAGGACCGCGCATC<br>TGCAAGGATGCTGGCGTAATGGTCACCAGCGA
TUB	TGACTCCTTCTTCAGGCAGACCATCTCTGGCGAGCACGGCCTTGACAGCAATGGCGTGTAC-<br>GTGATTGGTGCCGACCCTCGCCGCGGTTAGCCTCTCGCTCACTGGGTTCAATAGGTACAATGGCACCTCC<br>GAGCTCCAGCTCGAGCGCATGAACGTCTACTTCAACGAGGTGAGTT-<br>GCATCATGCCTTCCAACAGCCAGTCCGGAGGCGGGCTTGCTGACGGCGTTCTCTCTGCAGGCCTCCGGC<br>AACAAGTATGTCCCTCGTGCCGTTCTCGTCGACTTGGAGCCCGGCACCATGGAC-<br>GCCGTCCGCGCGGTCCCTTCGGCCAGCTCTTCCGCCCCGACAACTTCGTCTTTGGCCAATCCGGTGCCG<br>GCAACAACTGGGCCAAGGGTCA

Table S6. Sequence difference analysis.

Gene	Closest type strains	Sequence similarity(%)	Sequence difference(%)
LSU	*Achaetomium globosum* CBS 332.67	98.5	1.5
ITS		97.8	2.2
TUB	*Achaetomium strumarium* CBS 333.67	95.8	4.2

A

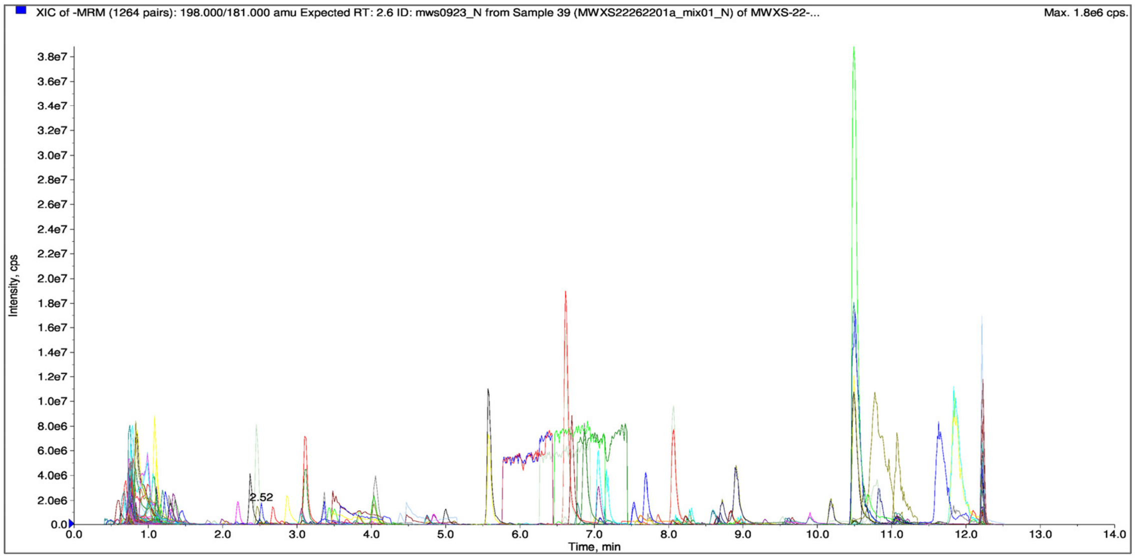

B

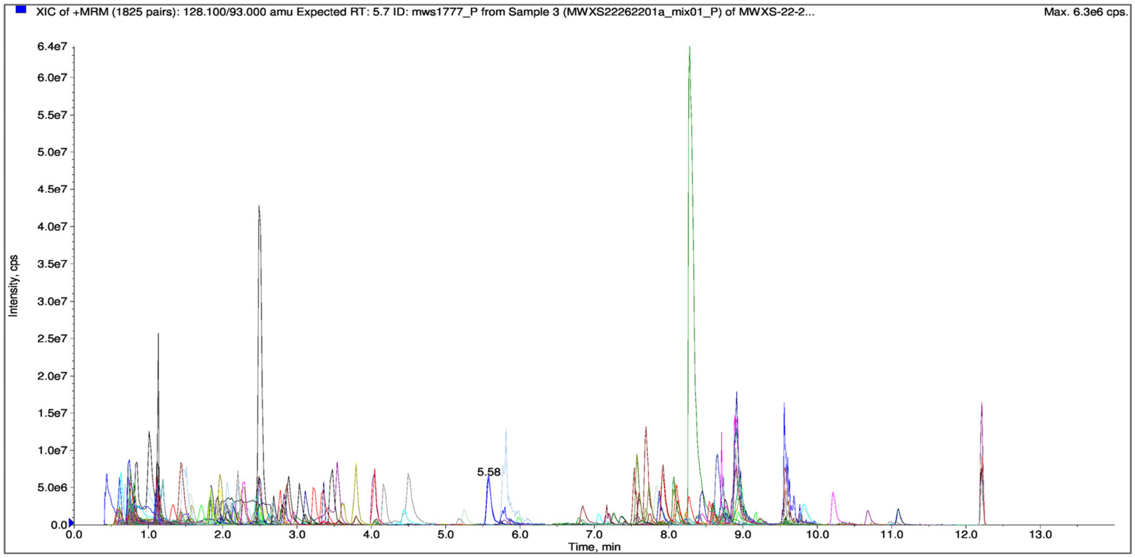

Figure S1. Antifungal substance mass spectrometry results. (A) Negative ion mode; (B) Positive ion mode.

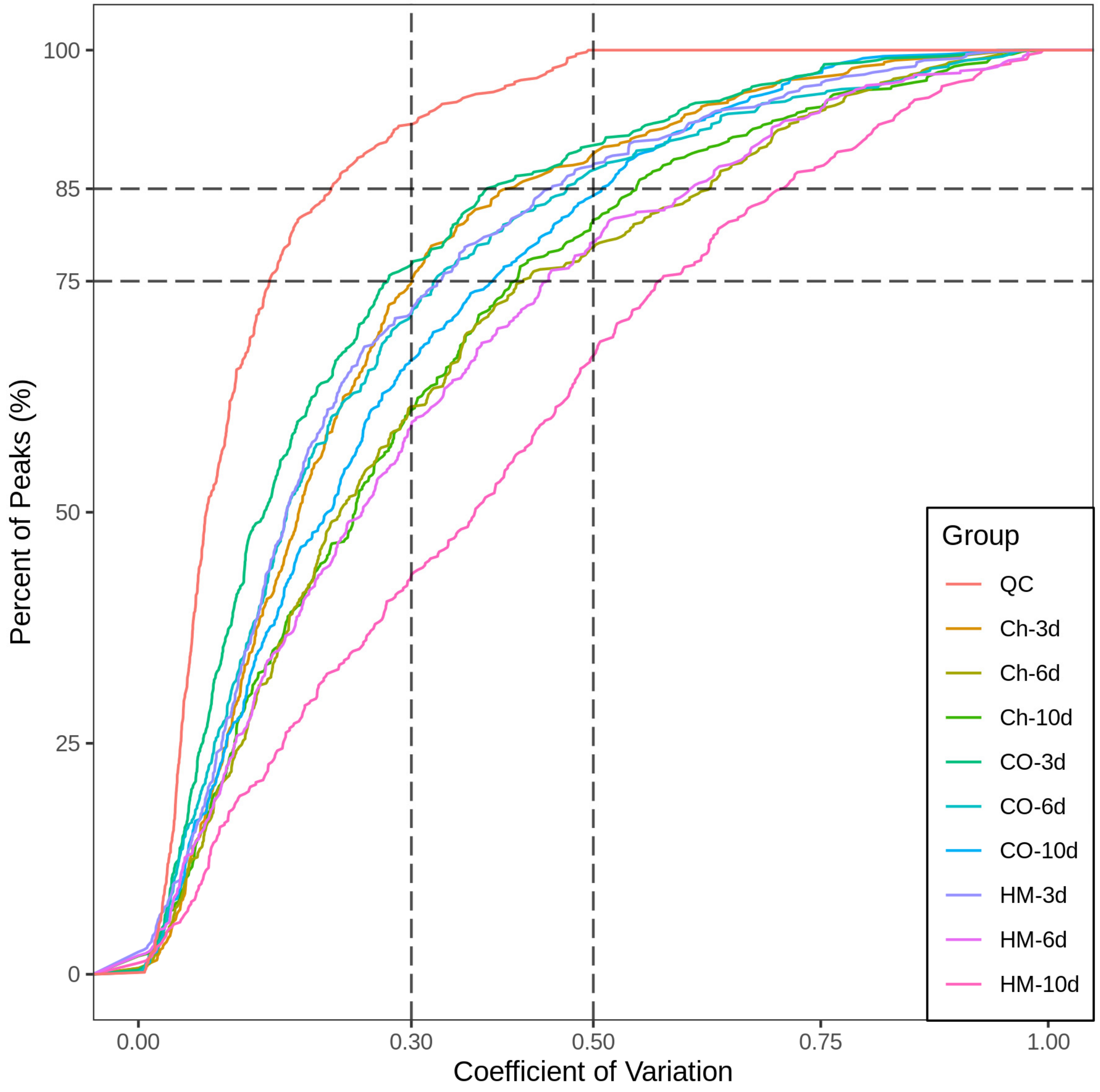

Figure S2. CV distribution diagram of each group of samples. The abscissa represents the CV value, and the ordinate represents the ratio of the number of substances less than the corresponding CV value to the total number of substances. Different colors represent different grouped samples, and QC is the quality control sample. The CV values corresponding to the two reference lines perpendicular to the X axis are 0.3 and 0.5, and the number of substances corresponding to the two reference lines parallel to the X axis accounts for 75% and 85% of the total number of substances.

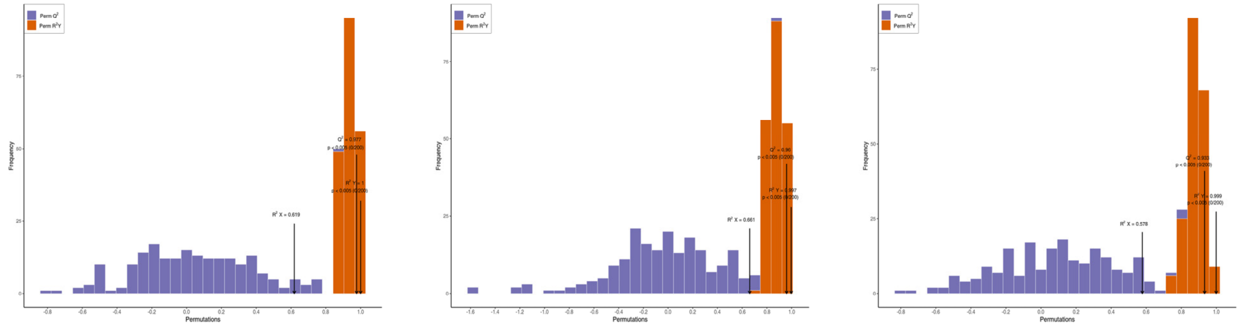

Figure S3. OPLS-DA permutation. The abscissa indicates the R^2Y and Q^2 values of the model, and the ordinate indicates the frequency of the model classification effect in 200 random permutation and combination experiments. In the figure, orange represents the random grouping model R^2Y, purple represents the random grouping model Q^2, and the values represented by the black arrows are R^2X, R^2Y and Q^2 of the original model. (From left to right, it is Ch-3d vs HM-3d vs CO-3d, Ch-6d vs HM-6d vs CO-6d and Ch-10d vs HM-10d vs CO-10d.)

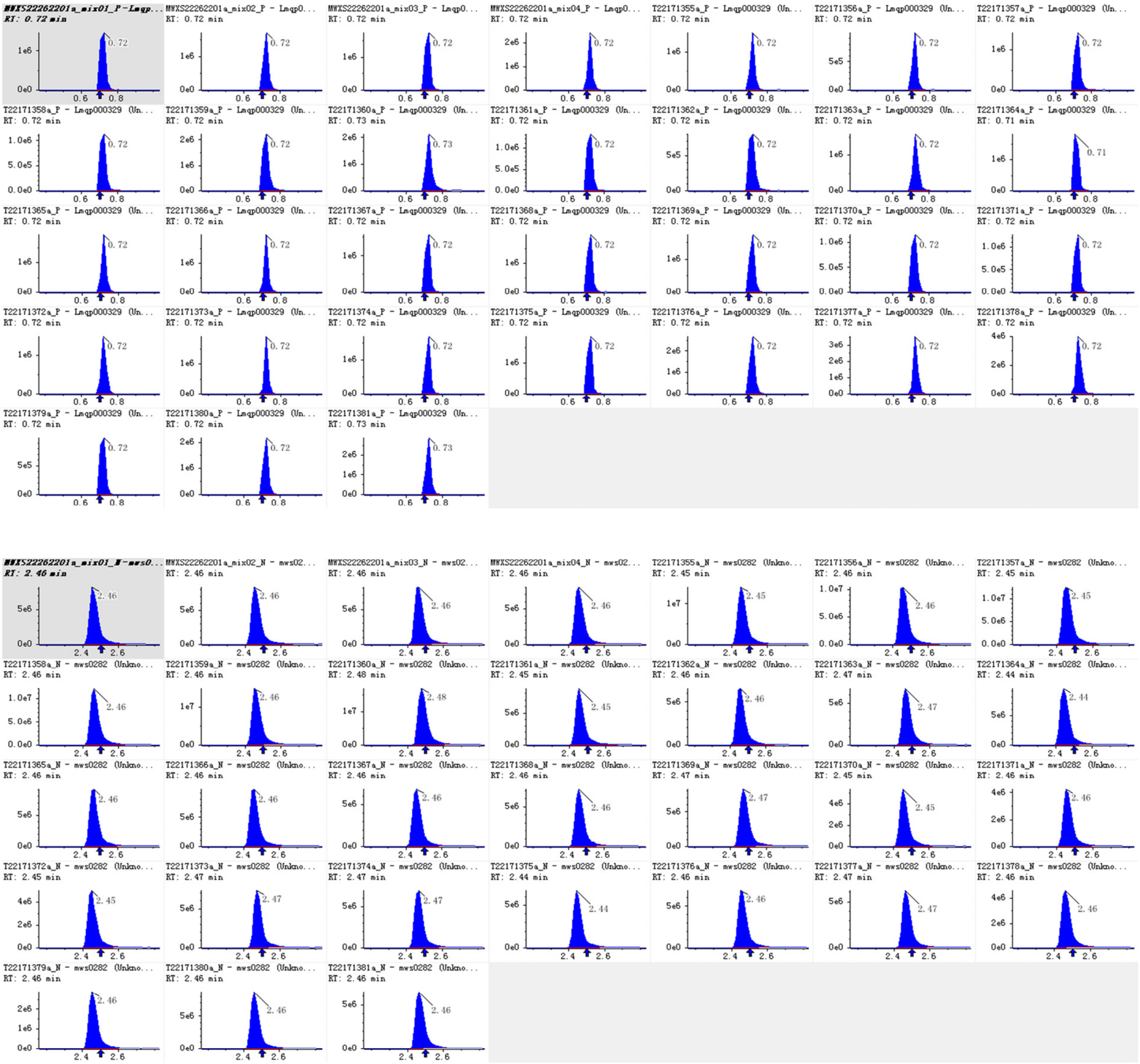

Figure S4. Integral correction chart for quantitative analysis of metabolites. Above is positive ion mode; The following is negative ion mode. The abscissa is the retention time (min) of metabolite detection, and the ordinate is the ion current intensity (cps) of a metabolite ion detection.

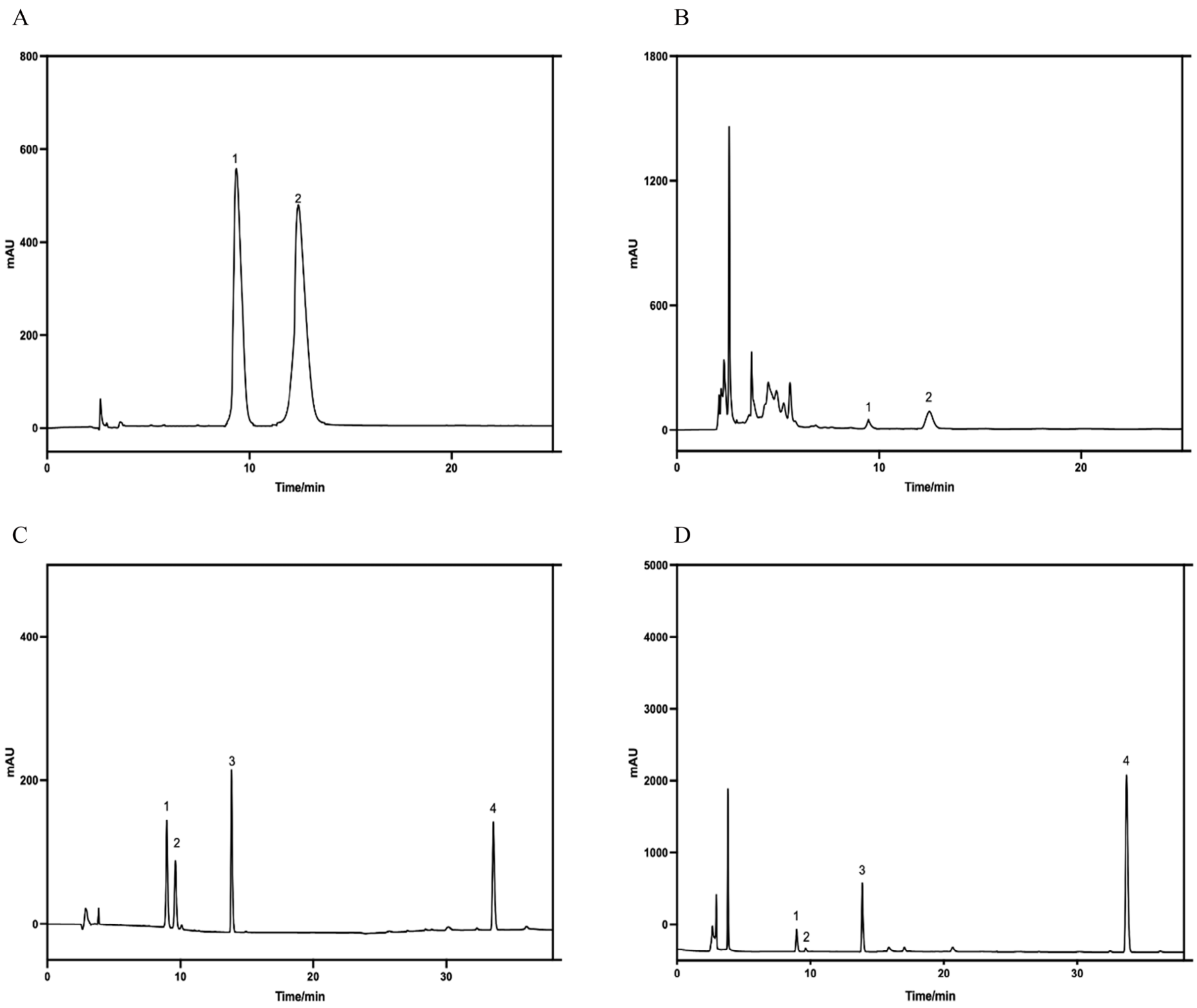

Figure S5. High-performance liquid chromatograms of three alkaloid and four flavonoid standards and mycelium extracts of A. sophora HY17. (A) Three alkaloid standards (1: Matrine; 2: Sophocarpine and Sophoridine); (B) Detection of alkaloids in hyphal extracts of A. sophora HY17; (C) four flavonoid standards (1: Vitexin; 2: Luteolin; 3: Daidzein; 4: Chalcone); (D) Flavonoid detection of hyphal extracts from A. sophora HY17.
